# Supplementary material for: Space–time dynamics of a triple epidemic: dengue, chikungunya and Zika clusters in the city of Rio de Janeiro
Source: Proc Biol Sci. 2019 Oct 9;286(1912):20191867. doi: 10.1098/rspb.2019.1867 (PMC6790786; doi:10.1098/rspb.2019.1867)
Supplement: Supplementary Material - Figures and Tables - Space-time dynamics of a triple epidemic: dengue, chikungunya, and Zika clusters in the city of Rio de Janeiro [file rspb20191867supp1.pdf]

Space-time dynamics of a triple epidemic: dengue, chikungunya, and Zika clusters in the city of Rio de Janeiro

Laís Picinini Freitas, Oswaldo Gonçalves Cruz, Rachel Lowe, Marilia Sá Carvalho

Proceedings of the Royal Society B

DOI: 10.1098/rspb.2019.1867

Supplementary Material

Figure S1. Rio de Janeiro city population density (inhabitants/m²) (A) and green areas (B), by region and neighbourhood, 2010.

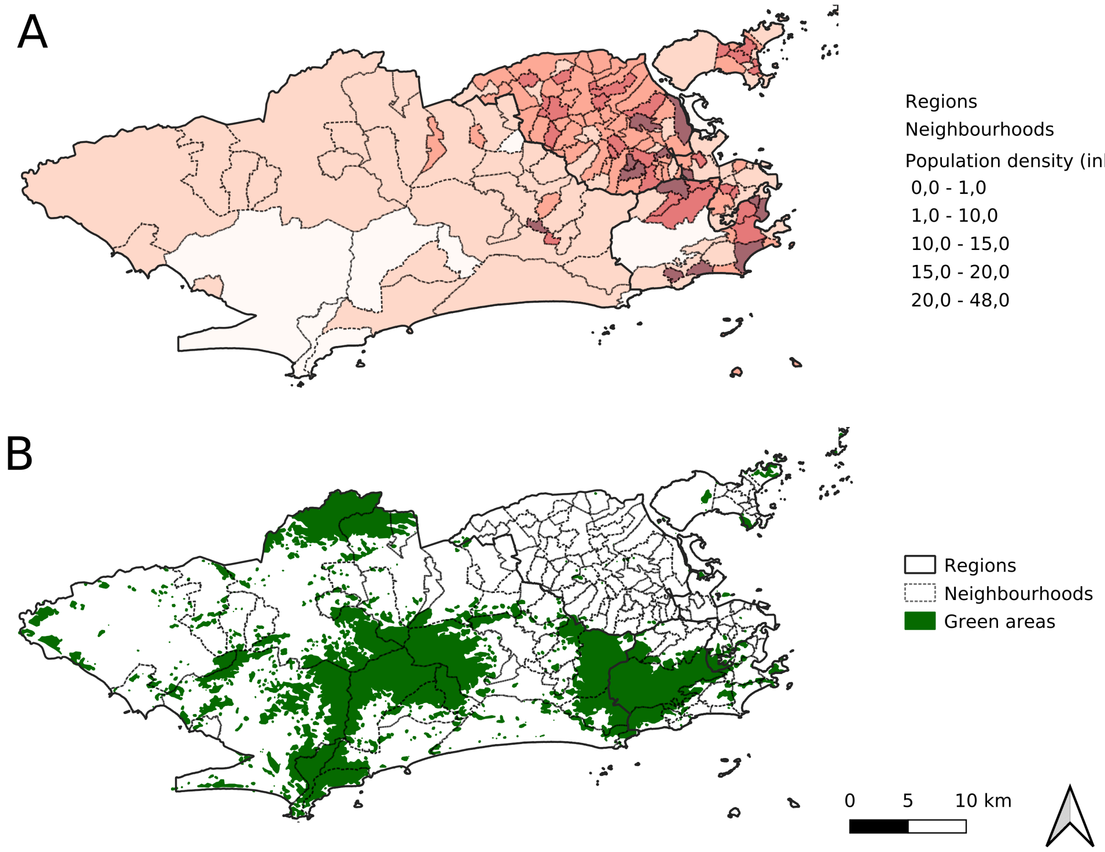

Maps were produced using QGIS (version 3.8.1). Sources: The Brazilian Institute of Geography and Statistics (IBGE) and Instituto Pereira Passos (IPP), Brazil.

Figure S2. Detection of Zika cases clusters in Rio de Janeiro city, Brazil, according to different temporal and spatial parameters using SaTScan™ (version 9.5), 2015-2016. A) Default parameters. B) Maximum temporal window of 1 week. C) Maximum temporal window of 4 weeks. D) Maximum temporal window of 4 weeks and maximum of 5% of population at risk. E) Maximum temporal window of 4 weeks and maximum of 1% of population at risk.

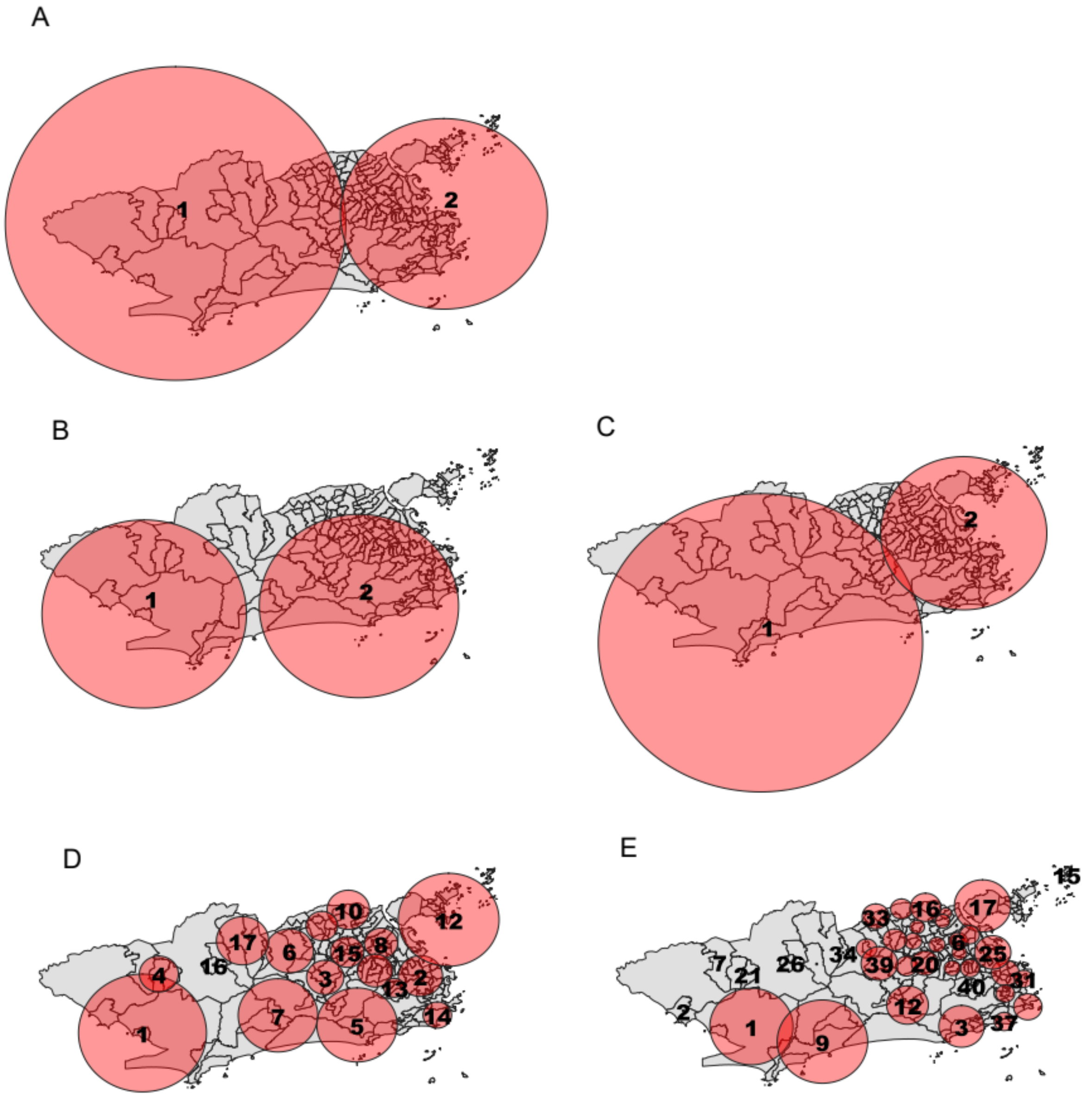

Note: This is the standard output of results from SaTScan™. A neighbourhood was considered part of a cluster if its centroid was inside the base of the cylinder (the circle, in this figure). Maps were produced using QGIS (version 3.8.1). Sources: Sistema de Vigilância de Agravos de Notificação (SINAN) – Ministry of Health, Brazil, and Instituto Pereira Passos (IPP), Brazil.

Table S1. Characteristics of dengue clusters between epidemiological weeks 31-2015 and 52-2016, Rio de Janeiro city, Brazil. Clusters are ordered according to the maximum log likelihood ratio, with 1 being the most likely cluster.

Cluster	Time period (week)	Population	Observed cases	Relative risk
1	10 to 14-2016	312654	1082	15.66
2	12 to 16-2016	12556	431	151.90
3	13 to 17-2016	296392	911	13.82
4	13 to 17-2016	243125	650	11.91
5	13 to 17-2016	105515	458	19.21
6	12 to 16-2016	290744	613	9.37
7	13 to 17-2016	283141	447	6.97
8	13 to 17-2016	278828	377	5.96
9	12 to 16-2016	304235	359	5.19
10	12 to 16-2016	217333	232	4.68
11	10 to 14-2016	313429	264	3.69
12	13 to 17-2016	94626	143	6.61
13	16 to 20-2016	225030	193	3.75
14	14 to 18-2016	3361	34	44.10
15	14 to 18-2016	315314	197	2.73
16	52-2015 to 4-2016	101443	89	3.83
17	12 to 16-2016	311869	151	2.11
18	12 to 14-2016	69356	29	3.64

Figure S3. Relative risks of clusters of (A) dengue, (B) chikungunya, and (C) Zika cases, detected between epidemiological weeks 31-2015 and 52-2016 in Rio de Janeiro city, Brazil.

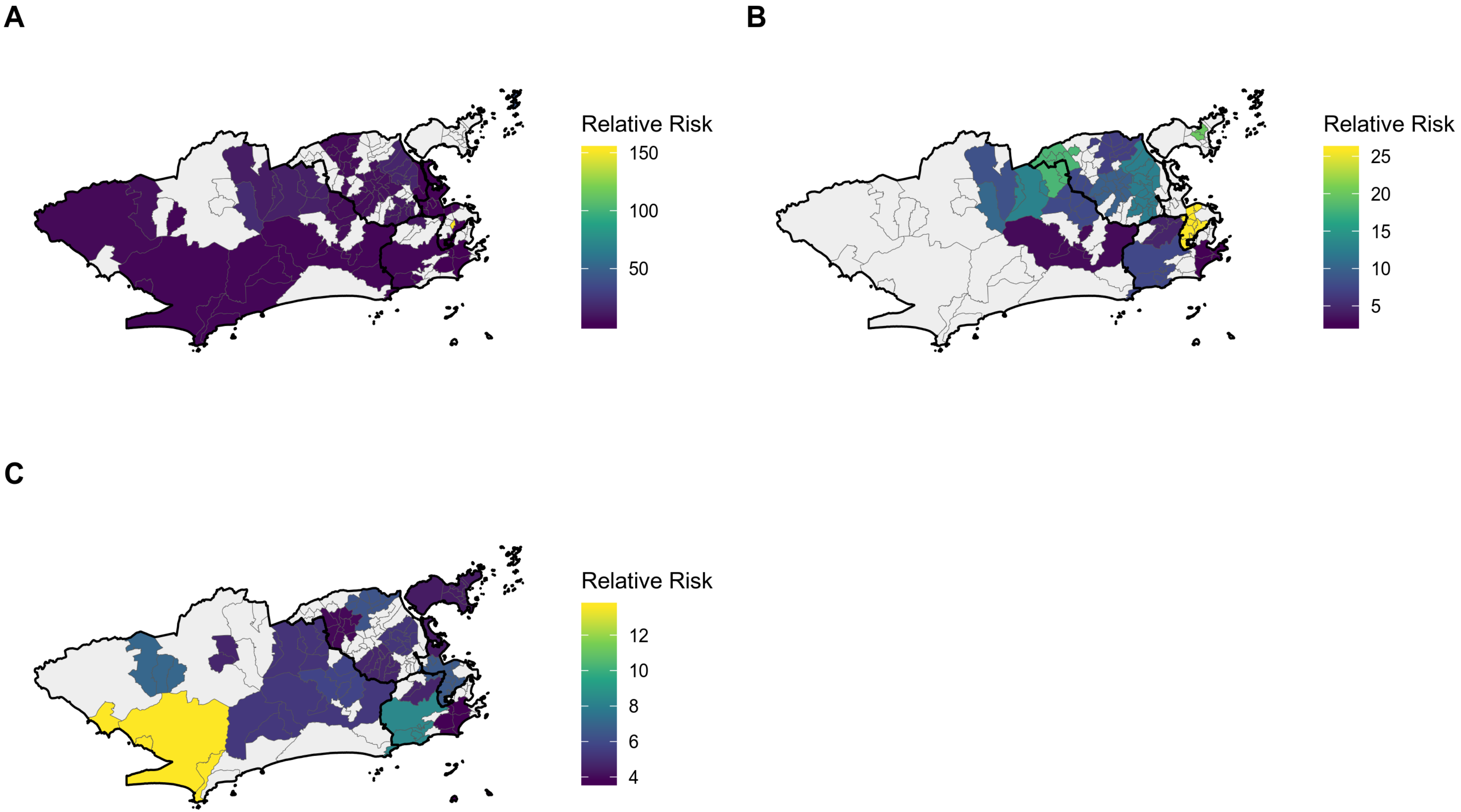

Maps were created using R (version 3.4.4) with ggplot2 package (version 3.1.0). Sources: Sistema de Vigilância de Agravos de Notificação (SINAN) – Ministry of Health, Brazil, and Instituto Pereira Passos (IPP), Brazil.

Figure S4. Week of cluster detection for (A) dengue, (B) chikungunya, and (C) Zika cases, in Rio de Janeiro city, Brazil. Red circles indicate the first cluster in time for each disease.

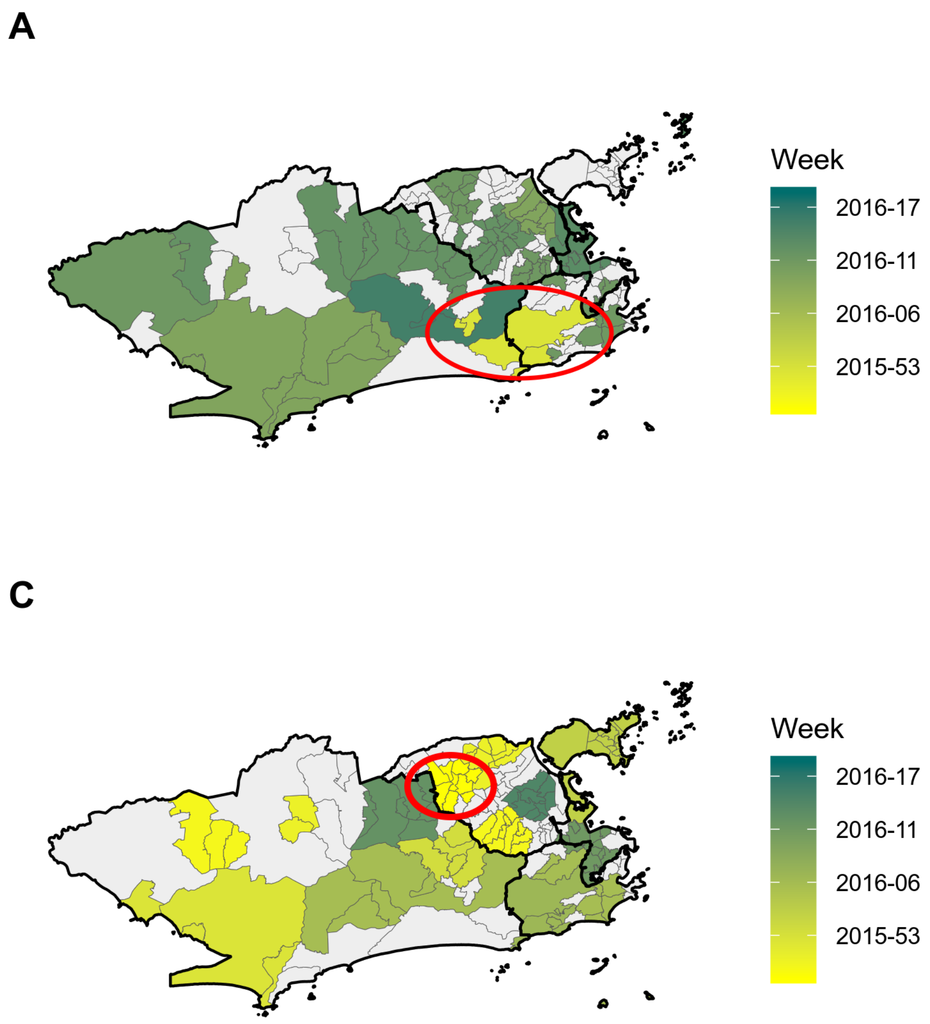

Maps were created using R (version 3.4.4) with ggplot2 package (version 3.1.0). Sources: Sistema de Vigilância de Agravos de Notificação (SINAN) – Ministry of Health, Brazil, and Instituto Pereira Passos (IPP), Brazil.

Table S2. Characteristics of chikungunya clusters between epidemiological weeks 31-2015 and 52-2016, Rio de Janeiro city, Brazil. Clusters are ordered according to the maximum log likelihood ratio, with 1 being the most likely cluster.

Cluster	Time period (week)	Population	Observed cases	Relative risk
1	13 to 17-2016	154001	453	25.77
2	12 to 16-2016	210786	434	18.00
3	16 to 20-2016	312654	448	12.53
4	16 to 20-2016	314738	442	12.28
5	16 to 20-2016	268781	397	12.87
6	14 to 18-2016	296540	332	9.71
7	19 to 23-2016	284673	256	7.75
8	15 to 19-2016	243125	237	8.40
9	16 to 20-2016	309599	243	6.76
10	16 to 20-2016	105515	128	10.38
11	16 to 20-2016	314444	171	4.66
12	16 to 20-2016	119297	105	7.52
13	19 to 20-2016	54415	32	19.99
14	19 to 23-2016	277454	98	3.01
15	17 to 20-2016	251142	57	2.57

Table S3. Characteristics of Zika clusters between epidemiological weeks 31-2015 and 52-2016, Rio de Janeiro city, Brazil. Clusters are ordered according to the maximum log likelihood ratio, with 1 being the most likely cluster.

Cluster	Time period (week)	Population	Observed cases	Relative risk
1	52-2015 to 4-2016	179689	742	13.57
2	49-2015 to 1-2016	236282	509	7.03
3	12 to 16-2016	247427	497	6.55
4	1 to 5-2016	309349	545	5.75
5	13 to 17-2016	307234	488	5.18
6	50-2015 to 1-2016	277724	404	6.31
7	7 to 11-2016	119297	307	8.36
8	49-2015 to 1-2016	294447	429	4.74
9	6 to 10-2016	231774	379	5.32
10	15 to 18-2016	297833	359	5.22
11	48 to 52-2015	298052	362	3.94
12	3 to 7-2016	233051	315	4.39
13	6 to 10-2016	203170	293	4.68
14	6 to 10-2016	306508	357	3.78
15	50-2015 to 2-2016	72058	105	4.71

Table S4. Characteristics of clusters of dengue, chikungunya, and Zika detected using multivariate scan statistic, between epidemiological weeks 31-2015 and 52-2016, Rio de Janeiro city, Brazil. Clusters are ordered according to the maximum log likelihood ratio, with 1 being the most likely cluster.

Cluster	Time period (week)	Population	Dengue relative risk	Chikungunya relative risk	Zika relative risk
1	12 to 16-2016	154001	21.16	25.30	7.66
2	13 to 17-2016	307234	13.50	8.04	5.18
3	10 to 14-2016	312654	15.66	2.86	3.27
4	12 to 16-2016	290744	9.37	6.81	4.11
5	52-2015 to 4-2016	179689	1.28	NA	13.57
6	13 to 17-2016	243125	11.91	5.32	1.18
7	14 to 18-2016	105515	19.04	7.93	1.93
8	13 to 17-2016	306968	6.27	8.86	3.58
9	12 to 16-2016	285585	5.22	6.84	3.90
10	49-2015 to 1-2016	236282	NA	NA	7.03
11	17 to 21-2016	309599	4.61	6.14	1.40
12	1 to 5-2016	301626	2.38	NA	4.98
13	7 to 11-2016	119297	1.09	NA	8.36
14	3 to 7-2016	233051	NA	NA	4.39
15	6 to 10-2016	306508	1.02	NA	3.78
16	50-2015 to 2-2016	72058	1.33	NA	4.71

Figure S5. (A) Minimum temperature (°C) and (B) precipitation (mm/month) in Rio de Janeiro city, Brazil, at the monthly time scale from January 2001 to December 2017.

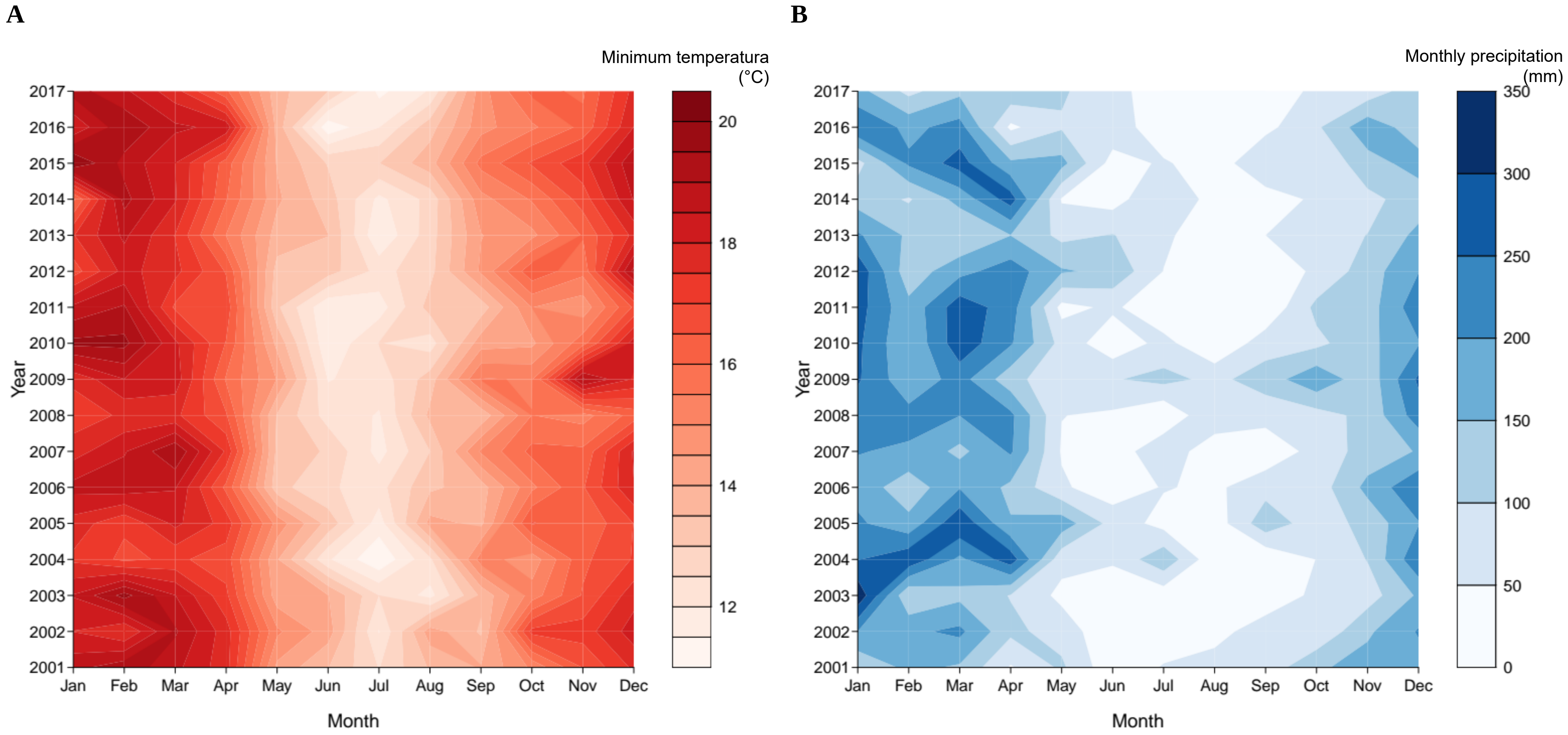

Figures were created using R (version 3.5.2). Source: Abatzoglou, J.T., S.Z. Dobrowski, S.A. Parks, K.C. Hegewisch, 2018, Terraclimate, a high-resolution global dataset of monthly climate and climatic water balance from 1958-2015, Scientific Data.
